# Characterization of Nuclear Mitochondrial Insertions in Canine Genome Assemblies

**DOI:** 10.3390/genes15101318

**Published:** 2024-10-14

**Authors:** Peter Z. Schall, Jennifer R. S. Meadows, Fabian Ramos-Almodovar, Jeffrey M. Kidd

**Affiliations:** 1 Department of Human Genetics, University of Michigan, Ann Arbor, MI 48109, USA; pschall@umich.edu (P.Z.S.); fabian.ramos-almodovar@pennmedicine.upenn.edu (F.R.-A.); 2 Department of Medical Biochemistry and Microbiology, Uppsala University, 75132 Uppsala, Sweden; jennifer.meadows@imbim.uu.se; 3 SciLifeLab, Uppsala University, 75132 Uppsala, Sweden; 4 Department of Computational Medicine & Bioinformatics, University of Michigan, Ann Arbor, MI 48109, USA

**Keywords:** canine, nuclear mitochondria insertion, Numt

## Abstract

Background: The presence of mitochondrial sequences in the nuclear genome (Numts) confounds analyses of mitochondrial sequence variation, and is a potential source of false positives in disease studies. To improve the analysis of mitochondrial variation in canines, we completed a systematic assessment of Numt content across genome assemblies, canine populations and the carnivore lineage. Results: Centering our analysis on the UU_Cfam_GSD_1.0/canFam4/Mischka assembly, a commonly used reference in dog genetic variation studies, we found a total of 321 Numts located throughout the nuclear genome and encompassing the entire sequence of the mitochondria. A comparison with 14 canine genome assemblies identified 63 Numts with presence–absence dimorphism among dogs, wolves, and a coyote. Furthermore, a subset of Numts were maintained across carnivore evolutionary time (arctic fox, polar bear, cat), with eight sequences likely more than 10 million years old, and shared with the domestic cat. On a population level, using structural variant data from the Dog10K Consortium for 1879 dogs and wolves, we identified 11 Numts that are absent in at least one sample, as well as 53 Numts that are absent from the Mischka assembly. Conclusions: We highlight scenarios where the presence of Numts is a potentially confounding factor and provide an annotation of these sequences in canine genome assemblies. This resource will aid the identification and interpretation of polymorphisms in both somatic and germline mitochondrial studies in canines.

## 1. Introduction

Since its origin as an organelle, components of the mitochondrial genome (mtDNA) have been repeatedly transferred to the eukaryotic nuclear genome [1]. Nuclear mitochondrial (Numt) sequences are segments of the nuclear genome with a recognizable origin from the mitochondrial genome [2]. Hybridization experiments from 40 years ago suggested the presence of mitochondrial-like sequences in the nuclear genome, and the advent of large-scale genome sequencing confirmed the ubiquitous presence of Numts across eukaryotes [3]. The analysis of diverse species indicates a wide range in the number and cumulative length of Numts across taxa, with some species showing evidence of post-insertion Numt amplification [2,4,5,6,7,8,9]. Numts can be useful markers for evolutionary studies and may show presence–absence dimorphism within a species [10,11,12,13,14]. Recent large-scale analyses in humans confirmed that Numt formation is an ongoing process, with new Numt insertions found in normal somatic tissues and tumors, as well as being transmitted through the germline [15,16]. The analysis of rare Numts in humans indicates that Numt breakpoints are enriched in non-coding segments of the mitochondria genome, Numt insertions are depleted near genes in the nuclear genome, and Numt formation involves multiple molecular mechanisms related to genome stability, DNA damage, and repair [15].

The presence of Numts in the nuclear genome complicates the analysis of mitochondrial sequence variation. Sequence differences between Numts and the mitochondria can be misinterpreted as mitochondrial mutations [17,18] or heteroplasmies [19,20,21,22]. Numts may also be associated with errors in genome assembly, resulting from false contig joins between nuclear and mitochondrial sequences that lead to the artifactual presence of large, mitochondrially derived sequences in an assembled nuclear chromosome [23,24,25].

Domestic dogs (*Canis lupus familiaris*) are a valuable model for studies of evolution and disease [26,27]. Similar to other mammals, dog genetic diseases caused by mutations in the nuclear genome are more widely studied than mitochondrial disease. However, multiple disease-causing mutations in dog mtDNA and affecting mitochondrial function have been described [28]. In canines, mitochondrial changes have also been associated with tumor progression [29,30] and the evolution of the clonally inherited canine transmissible venereal tumor (CTVT) [31,32,33]. Failure to account for Numts in the canine genome may confound the interpretation of canine mitochondrial variation.

Analysis of nuclear DNA from sperm heads [34], as well as the bioinformatic analysis of the initial canine genome assembly [35,36], confirmed the presence of Numts in dogs. Recent advances in long-read genome sequencing have led to the publication of multiple canine genomes, but the analysis of Numts across assemblies, including the characterization of dimorphic Numts, has been limited. As part of their assembly quality control procedure, Edwards et al. performed a systematic assessment of Numts in their basenji genome assemblies, finding patterns generally consistent with previous studies of Numts in mammals and limited dimorphism among assemblies [24]. To analyze mitochondrial variation in samples sequenced by the Dog10K Consortium, we previously identified large, high-identity Numts in the UU_Cfam_GSD_1.0/canFam4 assembly derived from a German Shepherd Dog named Mischka [37,38]. In this study, we provide a systematic analysis of Numts in 15 genome assemblies from dogs, wolves, and a coyote, and assess Numt sharing across Carnivora. We characterize multiple Numts that differ among assemblies and additionally identify dimorphic Numts using Illumina sequencing data from 1879 individuals. These data will aid future studies of somatic and germline mitochondrial variation in canines.

## 2. Materials and Methods

### 2.1. Identification of Numts in Genome Assemblies

We identified nuclear mitochondrial sequences (Numts) in canine genome reference assemblies based on the procedure previously used for analysis of the human genome [39]. First, the canine mitochondrial reference genome [40] (NC_002008.4) was searched against the genome assembly using the bl2seq functionality of blastn in the NCBI BLAST+ package [41], version 2.10.0. The search was performed with a scoring function of +2 for matches, −3 for mismatches, −5 for gap opening, and −2 for gap extension. Only high-scoring pairs (HSPs) with an e-value less than 0.001 were retained. HSPs with co-ordinates within 2000 bp in both the genome and mitochondria sequence, with consistent orientation, were merged into Numts regions (‘assembled Numts’ in the terminology of [39]). To refine HSP length and identity relative to the circular mitochondrial genome, a final set of HSPs was identified by searching the sequence of each merged Numt segment with a query consisting of the mitochondrial reference sequence concatenated twice. Numt length was tabulated based on the number of aligned mitochondrial bp in each HSP. The Numt content of 15 genome assemblies derived from 14 individuals was assessed (Table S1), including: two basenjis [24], two Bernese Mountain dogs [42], two different assemblies created from the same boxer [43,44], a Cairn terrier [42], a dingo [45], two German Shepherd Dogs [37,46], a Great Dane [47], a Labrador retriever [42], two wolves [48,49], and a coyote [49]. The analysis of the coyote assembly was performed using the coyote mitochondrial genome sequence [50] (NC_008093.1). Statistics were tabulated from Numt HSPs and merged Numts identified in each assembly, stratified by sequences assigned to assembled nuclear chromosomes (i.e., chr1-chr38 and chrX) or to unplaced contig sequences not assigned to an assembled chromosome. Alignments that span across the beginning and end of the linear mitochondria genome were represented as pairs of co-ordinates separated by commas.

### 2.2. Identification of Numt Differences Using Multiple Assemblies

Numts that differ between assemblies were identified by intersecting Numt HSPs with insertion and deletion variants found between assemblies. First, each assembly was aligned to the Mischka genome using minimap2 version 2.26 with option -x asm20 [51,52]. Insertion and deletion variants were identified using the paftools.js call command to identify variants in assembly segments covered by a single long alignment (-l 10,000 and -L 50,000 options). Insertions and deletions were converted to BED format and intersected with Numt HSPs annotated in each assembly using bedtools version 2.26.0 [53], reporting only variants that overlapped at least 90% of the Numt HSP (-f 0.90 option). The variant was classified as a large structural variant if the detected insertion or deletion was more than 1000 bp longer than the Numt HSP.

### 2.3. Comparison with Outgroup Genome Assemblies

To estimate Numt age, we searched for the presence of merged Mischka Numt loci in the arctic fox [54] (*Vulpes lagopus*, GCF_018345385.1), polar bear [55] (*Ursus maritimus*, GCF_017311325.1), and cat [56] (*Felis catus*, felCat9/GCF_000181335.3) genome assemblies. Each locus, along with 10 kbp of flanking sequence on each side, was extracted from the Mischka genome and searched against the polar bear and cat assemblies using BLAT [57] with options -stepSize = 5 -repMatch = 2253 -minScore = 20 -minIdentity = 0. The resulting hits were filtered to retain alignments that covered at least 2000 bp from the left and the right flanks, as well as at least 80% of the Numt. To confirm hits, candidate loci were extracted from the arctic fox, polar bear, and cat assemblies, and then queried against the dog mitochondrial sequence as described above.

### 2.4. Identification of Polymorphic Numts in Dog10K Samples

Structural variants identified and genotyped in 1879 dogs and wolves by the Dog10K consortium were retrieved from Meadows et al. [38]. Deletion structural variants were intersected with the merged Mischka Numt loci using bedtools, requiring a reciprocal overlap of 90% (-r 0.9).

Insertion variants were extracted from the VCF file, splitting by those completely assembled and those with only flanking left and right sequences. Complete sequences were converted to fasta format, using chromosome:position as identifiers. Incomplete sequences were joined with a string of 10 N’s between the left and right sequences and outputted to fasta format. The two resultant fasta files were concatenated and analyzed using numtfinder [24] to identify non-reference Numts, using the NC_002008.4 mitochondrial sequence, with the addition of the circle = T flag. The number of Numts present per sample was determined based on the genotypes in the Dog10K VCF file and summarized by category including breed dogs, dogs of mixed origin, or that are not recognized by any international registering body (labeled as Mixed/Other), village dogs, and wolves.

## 3. Results

### 3.1. Identification of Nuclear Mitochondrial Sequences in the Mischka UU_Cfam_GSD_1.0/canFam4 Genome Reference

**Figure 1:**
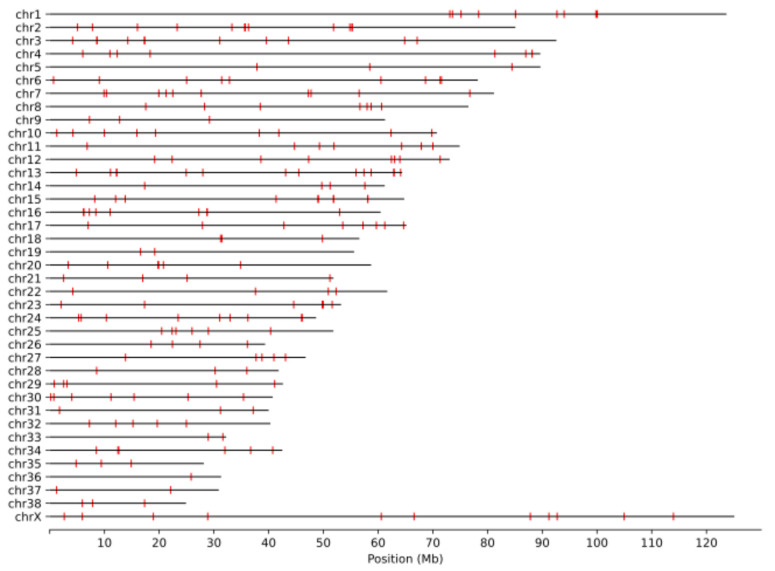
Position of Numts in the Mischka genome. The position of 243 merged Numt loci along the assembled nuclear chromosomes in the Mischka (UU_Cfam_GSD_1.0/canFam4) genome is shown. The location of each Numt is indicated by a red box.

**Figure 2:**
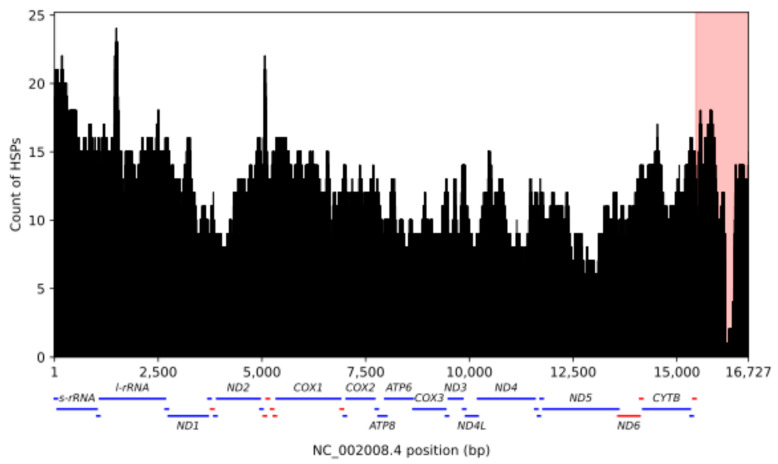
Coverage of canine mtDNA along the assembled Mischka genome. The total coverage along the mitochondria is shown for the 316 Numt HSPs identified in the assembled chromosomes from Mischka. The shaded pink region corresponds to the mitochondrial D-loop. The position of mitochondrial genes on the forward (blue) and reverse (red) strand is depicted below the figure. The names of rRNA and protein-coding genes are given.

The UU_Cfam_GSD_1.0 assembly [37], derived from a German Shepherd Dog named Mischka and labeled as canFam4 in the UCSC Genome Browser, has emerged as the main reference used for the analysis of canine genome variation [38]. We therefore first annotated nuclear mitochondrial sequences (Numts) in the Mischka assembly following the procedure previously used for the analysis of humans and other species [39]. We identified a total of 321 Numt HSPs, with five HSPs located on assembled contigs that are not integrated into the chromosomal-level assembly (Table 1, Tables S2 and S3). This includes a full-length copy of the mitochondria genome with high sequence identity that makes up chrUn_JAAHUQ010000987v1 and is likely the result of assembly error. We identified 316 Numt HSPs encompassing 200,108 mitochondrial bp on the assembled chromosomes. These 316 segments can be merged into 243 Numt loci which are located across all 38 autosomes and the X chromosome (Figure 1). A similar distribution of Numts was found across 14 other canine genome assemblies (Tables S2 and S3, and Figure S1). The Numt segments encompass the entire mitochondrial genome, with reduced coverage found in the D-loop which has been previously found to be depleted in Numts across primates and which contains a short repeat sequence that is highly variable in canines [39,58] (Figure 2).

It is not straightforward to compare the raw counts of annotated Numt HSPs between genomes. For example, the longest Numt HSP identified in the Mischka assembled chromosomes is found on chr34, encompasses 10,195 mitochondrial bp with an identity of 83.14%, and is also present in the mCanLor1.2 wolf genome. However, in mCanLor1.2, this long Numt is disrupted by a LINE-1 insertion that occurred after Numt integration (Figure S2) and is therefore reported as separate HSPs.

### 3.2. Dimorphic Numts between Mischka and Other Genome Assemblies

**Figure 3:**
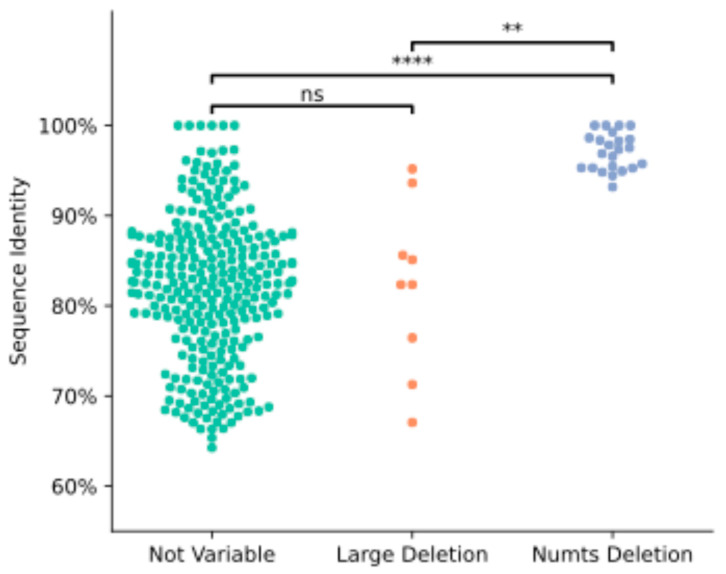
Dimorphic Numts have a higher mitochondrial sequence identity than fixed Numts. A swarm plot of sequence identity relative to the mitochondrial reference genome is shown for Numts annotated in the Mischka assembly, values are plotted for 284 Numts that are found in each assembly, 9 Numts that overlap larger deletions, and 23 Numts that are absent in other assemblies. Sequence identities were compared across categories using Welch’s unequal variances *t*-test: ns: not significant, ** *p* < 0.01, **** *p* < 0.0001.

We searched for Numts that have presence–absence dimorphism between Mischka and other genome assemblies from dogs, wolves, and a coyote. First, we identified a total of 32 Numt HSPs (14,528 total bp) annotated in Mischka that appear to be absent in another assembly (Table S4). This includes 9 Numt HSPs that overlap with larger structural variants identified between assemblies and 23 Numts that overlap with deletion variants with a length within 1 kbp of the Numt size. Of the 23 cleanly dimorphic Numts, 6 were absent only in the coyote. One Numt, a 195 bp HSP with 98.5% identity, was absent in every other assembly analyzed. As expected, the dimorphic Numts have a higher identity to the reference mitochondria than those which are fixed in all samples (*p* < 0.0001, Figure 3). Considering more distantly related members of the carnivore linage, 169 of the 243 merged Numts present on assembled chromosomes in Mischka are found in the arctic fox (*V. lagopus*) genome while 20 are present in the polar bear (*U. maritimus)* genome and 8 are present in the cat (*F. catus*) genome, indicating that a subset of Numts have an ancient origin dating to the initial diversification of the order Carnivora (Table S5).

We additionally searched for Numts that appear as insertions in the other assemblies relative to Mischka and identified 31 loci (Table S6). Six of these correspond to large structural variants that include a Numt sequence, while 25 are insertion differences corresponding to Numts. These 25 loci include five Numts that were found only in the coyote, as well as a 3280 bp segment present only in Sandy, the dingo. Together, the analysis of these assemblies identifies 63 Numts that show presence–absence dimorphism among dogs, wolves, and coyotes.

### 3.3. Comparison with 1879 Samples Analyzed by the Dog10K Consortium

The Dog10K consortium identified structural variants in 1879 diverse canines based on alignment of Illumina sequencing data to the Mischka genome assembly [38]. The Dog10K deletions include 11 variants that have a reciprocal 90% overlap with a merged Numt locus annotated in Mischka (Table S7). This includes one Numt not identified as variable based on the analysis of 14 genome assemblies described above.

**Figure 4:**
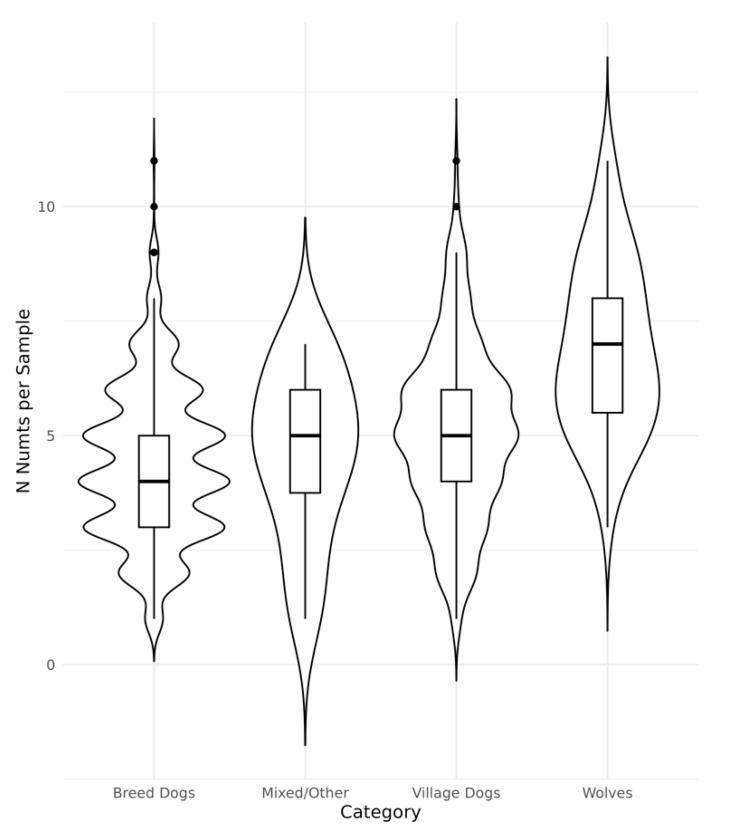
Numts insertions identified in Dog10K samples. Insertions identified in 1879 canines by the Dog10K consortium were analyzed to identify 53 insertions that correspond to Numts. Violin plots and boxplots are shown of the number of Numts present per sample, stratified by category: Breed Dogs (*n* = 1575), Mixed/Other (*n* = 12), Village Dogs (*n* = 237) and Wolves (*n* = 55).

The Dog10K structural variant collection also includes insertions along with assemblies of the insertion sequence. Since the Dog10K data is derived from Illumina reads, the full sequence of large insertions (⪆200 bp) could not be resolved and is represented as partial segments extending into the variant from each edge. We compared the reported sequence of each insertion with the mitochondrial reference and identified 53 insertions that correspond to Numts (Table S8). This includes seven variants where the insertion sequence is only partially assembled. Of the 53 insertions, 15 were also identified in our analysis of the 14 additional genome assemblies. Dog10K samples contained a median of four Numts, with a range of 0 to 11 (Figure 4 and Figure S3). Of the 1879 samples, only 12 did not have any of the 53 insertions. Delineating by canine category, Breed Dogs (*n* = 1575) displayed a median of four insertions per sample (range = 0–11), Village Dogs (*n* = 237) median of five (range = 1–11), and Wolves (*n* = 55) with a median of seven (range = 3–11).

### 3.4. Numts and Genome Annotations

To assess the intersection of Numts with gene annotations, we created a merged list of all Numts identified in the Mischka reference, other canine genome assemblies, and in the Dog10K samples. Numts with genomic co-ordinates within +/− 10 bp were considered the same, resulting in a total of 310 regions. Of these, 162 are located in intergenic regions and 148 are present in introns with 137 different genes containing an intronic Numt (Table S9). Three Numts annotated in the Misckha reference overlap with CpG islands (chr2:33295939-33296111, chr4:88140443-88140514, and chr4:88140516-88140592), as well as a Numt at chr25:49761457 that was found in China, Cla-1, Wags, mCanLor1.2, and the Dog10K dataset.

## 4. Discussion

The Mischka genome [37] (UU_Cfam_GSD_1.0/canFam4), derived from a German Shepherd Dog, has emerged as a common reference genome used for studies of canine variation, making annotation of Numts in this genome a valuable resource for the canine genomics community [38]. We identified 321 Numt HSPs in the Mischka genome that in total encompass the entire mitochondrial genome sequence. This includes a full-length representation of the mitochondria genome present on an unplaced sequence that is included in the Mischka assembly but is not localized to an assembled chromosome.

The representation of mitochondrial sequences in canine Numts is not uniform (Figure 2). The sharp reduction in the D-loop region corresponds to a minisatellite repeat that is highly variable in canines [59,60]. A general pattern of reduced representation across the mitochondrial D-loop region has been reported previously [58], while a recent survey suggests the distribution of segments included in Numts may vary across species [14]. A large-scale analysis of inherited, de novo, and somatic Numts in humans showed that somatic Numts present in tumors are more likely to include the D-loop than Numts present in the germline [15]. Thus, the observed representation of mitochondrial segments may reflect a combination of differences in the intrinsic insertion propensity of segments, post-insertion selection, and bias in discovery due to higher sequence diversity in the D-loop [61,62].

Although the impact of Numts on canine phenotypes is unclear, studies in humans have associated several Numts with disease, including insertions of mitochondrial sequences into protein coding genes [63,64] and incorporation of mitochondrial sequences at rearrangement breakpoints during DNA double-strand break repair [65]. Numts may also contribute to somatic variation. Somatic Numts have been observed in tumors and brain tissue in humans [15,16]. Numts have also been associated with altered DNA replication dynamics and chronological ageing in yeast [66,67,68].

Knowing the location and types of Numts within the analyzed reference sequence is key to the success of future research projects. From a nuclear genome perspective, a Numt exclusion list can highlight those regions likely to negatively impact short read mapping or cell-free mtDNA detection [3,35,69,70]. From the mitogenome perspective, if not accounted for, the full-length Numt spanning an unplaced contig in the Mischka genome will act as a decoy and siphon reads from the true mitochondrial genome. This has the potential to lead to underestimates of mitochondrial copy number and to reduce the measurable allele fraction of true mitochondria variants. Additionally, false levels of mitochondrial heteroplasmy will be estimated if Numt sequences are not accounted for in mitochondrial mapping and variant calling pipelines. This problem can be addressed through the incorporation of a Numt reference list to mitochondrial mapping software. To facilitate such analyses, we have collated the Numt locations in the Mischka, Tasha, CanFam3.1, Zoey and mCanLor1.2 genomes into an UCSC Genome Browser Track Hub [71,72].

To put our annotation of Mischka into a larger context, we identified Numts in 14 other genome assemblies. Previous analysis showed that canine genome assemblies differ markedly in the representation of duplicated sequences on unplaced chromosome contigs [73]. The same is true for Numts: three assemblies show substantial Numt representation on unlocalized contigs while the others contain little or no Numt sequence on unplaced contigs. These discrepancies likely reflect differences in the assembly algorithms and filtering strategies employed for each genome.

Of the Numts annotated in Mischka, 32 were absent in at least one other canine genome assembly. Since genomic DNA is generally not available from the samples used to construct each assembly, we were not able to experimentally validate the annotated Numts. However, we note that the dimorphic Numts have a higher sequence identity with the reference canine mitochondria genome, consistent with a more recent origin. The analysis of a diverse collection of 1879 canines additionally identified 11 Numts loci that are deleted in at least one sample, as well as 53 insertions that correspond to non-reference Numts. Together, these observations indicate that some Numts formed recently in canine evolution and have not become fixed in canines. The presence of polymorphic Numts is a confounding factor that must be accounted for in studies of mitochondrial sequence variation, mitochondrial heteroplasmy, and somatic mutation in canines.

## Figures and Tables

**Table 1:** Summary of Numts identified in genome assemblies from a dog, wolf, and coyote.

Assembly Name	Mischka	mCanLor1.2	Cla-1
Sample Type	German Shepherd Dog	Greenland wolf	coyote
*Assembled Chromosomes*			
HSP Count	316	312	316
Total Size (bp)	200,108	193,976	195,514
Max Length (bp)	10,195	6527	10,197
Median Length (bp)	173.5	174	159.5
Merged Loci	243	238	240
*Unplaced Contigs*			
HSP Count	5	0	0
Total Size (bp)	23,275	0	0

## Data Availability

A UCSC Track Hub of Numt locations is available at https://github.com/KiddLab/dog-numts-tracks (accessed on 12 September 2024). The script used for Numt HSP identification and merging is available at: https://github.com/jmkidd/refNUMTS (accessed on 12 September 2024).

## Supplementary Materials

The following supporting information can be downloaded at: https://www.mdpi.com/article/10.3390/genes15101318/s1, Figure S1: Numts identified per chromosome; Figure S2: A long Numt is disrupted by a LINE-1 insertion in the mCanLor1.2 assembly; Figure S3: Allele frequency of 53 Numt insertions identified in Dog10K samples; Table S1: Analyzed genome assemblies; Table S2: Summary of Numts identified in 15 analyzed assemblies; Table S3: Numts coordinates in all analyzed assemblies; Table S4: Summary of Mischka Numts deleted in other assemblies; Table S5: Summary of Numts sharing in other carnivores; Table S6: Numts identified in other assemblies that are absent in Mischka; Table S7: Numts in Mischka that correspond to deletions identified by the Dog10K consortium; Table S8: Insertions identified by the Dog10K consortium that correspond to Numts; Table S9: Annotation of Numt genic regions.
